# Chronic rhinosinusitis with and without nasal polyps and asthma: Omalizumab improves residual anxiety but not depression

**DOI:** 10.1002/clt2.12002

**Published:** 2021-03-15

**Authors:** Florian Vogt, Jagdeep Sahota, Therese Bidder, Rebecca Livingston, Helene Bellas, Simon B. Gane, Valerie J. Lund, Douglas S. Robinson, Harsha H. Kariyawasam

**Affiliations:** ^1^ Department of Respiratory Medicine University College London Hospital NHS Foundation Trust London UK; ^2^ Rhinology UCL Ear Institute University College London London UK; ^3^ Rhinology Section Royal National ENT Hospital London UK

**Keywords:** anxiety, asthma, depression, omalizumab, rhinosinusitis

## Abstract

**Background:**

Chronic rhinosinusitis (CRS) has a high prevalence of anxiety and depression. It is currently uncertain if treatment in patients with CRS with or without nasal polyps (CRSwNP and CRSsNP) has any impact on improving mental health outcomes. The aims here were to document anxiety and depression in patients with severe CRS and asthma already treated with appropriate medical therapy. We then evaluated whether further maximal treatment with omalizumab improved anxiety and/or depression alongside improvements in CRS and coassociated asthma.

**Methods:**

Hospital Anxiety and Depression Scale (HADS) scores along with measures of CRS and asthma severity were recorded according to CRSwNP and CRSsNP status in *n* = 95 patients with severe CRS and asthma. Of this group, a further *n* = 23 had omalizumab for associated allergic asthma. Follow‐up measures were collected 16 weeks after omalizumab treatment.

**Results:**

HADS anxiety and depression prevalence in CRS were 49.47 % and 38.95%, respectively. Within the CRSwNP and CRSsNP group 53.06% and 45.66% had raised HADS‐anxiety scores. Abnormal HADS‐depression scores were present in 40.82% and 36.95% of the CRSwNP and CRSsNP groups, respectively. Correlations for sinonasal outcome test‐22 (SNOT‐22) versus HADS total was *r* = 0.59 *p* < 0.0001, HADS‐anxiety *r* = 0.56 *p* < 0.0001 and HADS‐depression *r* = 0.49 *p* < 0.0001. Omalizumab improved anxiety in CRS (*p* < 0.0001) regardless of nasal polyp status (CRSwNP *p* = 0.0042 and CRSsNP *p* = 0.0078). Depression scores did not improve in either group. SNOT‐22 (*p* = 0.0006), asthma control questionnaire‐7 (*p* = 0.0019) and mini‐asthma quality of life questionnaire including emotional function (*p* = 0.0003 and *p* = 0.0009, respectively) all improved in both subgroups.

**Conclusion:**

In CRS and asthma, anxiety scores but not depression improved after omalizumab treatment. Anxiety may be closely related to airway disease severity, but depression may be independent of airway disease itself. If so, a separate mental health care pathway is needed for CRS patients with depression.

## 1 BACKGROUND

Chronic rhinosinusitis (CRS) is the broad term used to define sinonasal inflammation of at least 12 weeks duration.^1^ It is estimated to affect around 10%–15% of Europe and US populations. The prevalence of poor mental health in CRS varies across studies but estimated to be as high as 17%–32% for anxiety^2^, ^3^, ^4^ and 11%‐40% for depression.^5^ In a Taiwanese database cohort study, depression in CRS was a staggering 77% higher compared to non‐CRS controls.^6^

Poor mental health has significant implications for not only patient well‐being but also healthcare utilisation. The treatment cost of CRS has been estimated at $64.5 billion US dollars per year.^7^ Anxiety and depression can alter perception of disease and thus lead to symptom amplification and increased distress.^8^ ^,^ ^9^ Such factors may explain why objective disease findings on nasal endoscopy and sinus CT scoring often correlate poorly with the actual levels of subjective sinonasal symptoms.^10^ In addition, compromised mental health can lead to poor adherence with medication^11^ and increased health‐seeking behaviour.^12^ Despite frequent hospital attendance, as high as 24 million patient visits per year in the United States^7^ and often overtreatment,^3^ ^,^ ^13^ treatment outcomes are often suboptimal^13^ resulting in dissatisfied patients, frustrated health‐care workers and escalating costs of treatment. Thus, it is important to understand what disease factors cause poor mental health in CRS, and importantly determine whether actual treatment of disease can improve anxiety and depression in CRS patients.

Despite numerous studies, it is still unclear whether CRS severity contributes to anxiety and depression. There is conflicting literature on whether treatment of CRS improves mental health status. Small size and methodological limitations of such studies reporting outcomes in heterogeneous CRS populations limit their interpretation. Current appropriate medical therapy (AMT) for CRS is primarily nasal douching and intranasal steroids.^1^ The other separate or combined option is endoscopic sinus surgery (ESS). In a database of 15,371 patients, depression in CRS was present to the same degree regardless of previous surgical intervention.^6^ Studies have shown surgical intervention for CRS does not improve mental health^14^ and patient depression is associated with poorer postsurgical outcomes.^2^ ^,^ ^15^ In contrast, a separate study showed that both AMT and ESS improved depression.^13^ One difficulty when assessing CRS medical intervention studies is that AMT for more severe CRS is often ineffective.^1^ AMT can also involve medications with common adverse events such as long‐term antibiotics and systemic steroids. Thus, any improvement in CRS contributing to better mental health could be counteracted by medication‐related adverse events. For example, oral prednisolone can both cause and exacerbate neuropsychiatric dysfunction. Increased ocular, metabolic, bone and cardiovascular‐related complications are common.^16^ ^,^ ^17^

The role of commonly associated comorbidities should be considered. CRS and severe asthma are often associated^18^ and anxiety and depression are also common in asthma populations. Pooled data from the World Mental Health Survey showed that the age and gender‐adjusted odds for anxiety and depression in asthma versus nonasthma was 1.5 (95% confidence interval [CI]: 1.4–1.7) and 1.6 (95% CI: 1.4–1.8), respectively.^19^ It has been shown that the prevalence of depression in CRS remains high even when corrected for asthma comorbidity.^20^ In studies assessing whether treatment of CRS improves mental health, coassociated asthma is not well controlled for or maximally treated. Thus, it is still possible that symptoms from lower airway disease drive poor mental health status in CRS.

In this study we report the prevalence of anxiety and depression in a tertiary rhinology centre. All patients had primary diffuse bilateral CRS^1^ with either CRS with nasal polyps (CRSwNP) or CRS without nasal polyps (CRSsNP) and associated severe asthma. All patients had undergone medical or surgical treatment or both for CRS and as well as having effective asthma management. We also evaluated whether anxiety and depression were reduced in a subgroup of such patients when given omalizumab (anti‐IgE) for severe asthma with associated CRS.

## 2 METHODS

### 2.1 Recruitment

Patients with CRS that attended a tertiary rhinology centre over a 3‐year period underwent detailed combined review by an ear, nose and throat surgeon (ENT) and pulmonologist. CRS subtype was confirmed by sinonasal endoscopy with computer tomography sinus if needed. Guideline‐based management with AMT for CRS (nasal douching, intranasal‐steroid and if indicated an immune‐modulatory antibiotic) and associated asthma with/without ESS was started. Patients who continued to have poorly controlled or severe asthma were then further evaluated through a multidisciplinary team difficult asthma assessment protocol. Here, baseline mental health status was reviewed by a clinical psychologist and measures of upper and lower disease were recorded prior to any further intervention. All patients with potential vocal cord dysfunction and primary headache‐facial pain were excluded from the study. The subgroup of severe asthma patients that remained poorly controlled and qualified for omalizumab based on UK prescribing criteria (https://www.nice.org.uk/guidance/ta278), were treated and assessed in terms of mental health status alongside improvements in pan‐airway function after 16 weeks of omalizumab treatment.

### 2.2 Instruments

The Hospital Anxiety and Depression Scale (HADS) 14‐item assessment tool was used as a measure of mental health status. It is the UK National Institute of Clinical Excellence (https://www.nice.org.uk/guidance/cg123) recommended assessment tool for anxiety and depression. Based on seven assessment items for anxiety (score range: 0–21) and seven for depression (score range: 0–21), they can be looked at as total HADS and separately as HADS‐anxiety and HADS‐depression. Higher the scores then greater the psychological burden. A score of 0–7 is classed as normal range, 8–10 borderline abnormal and 11–21 abnormal for each parameter.^21^

The sinonasal outcome test‐22 (SNOT‐22) score is a subjective measure of CRS severity and provides information on both CRS symptoms and its burden on general well‐being. With a total possible score of 110, severity is defined as mild when 8–20 inclusive, moderate when more than 20–50 and severe when more than 50.^22^

The asthma control questionnaire‐7 (ACQ‐7) is comprised of seven items in relation to asthma symptoms and rescue inhaler use with measured forced expiratory volume in one second percentage predicted (FEV_1_%) on the day of completion. With complete asthma control the score is 0 and when severely uncontrolled is 6.^23^

The asthma quality of life questionnaire (AQLQ) has a total of 32 questions comprised of four domains (symptoms, activity limitation, emotional function and environmental stimuli). There is a 7‐point scale where seven is taken as no impairment and a score of one is severely impaired. The average score of all 32 responses is the final AQLQ score.^24^

The mini‐AQLQ is a shorter version that based on capturing quality of life (QOL) in relation to asthma based on five items on symptoms, four on activity limitations, three on emotional function and three environmental stimuli.^25^ Given time constraints during omalizumab injection related clinic visits, the mini‐AQLQ was used instead of the AQLQ.

### 2.3 Statistical analysis

Demography and clinical traits for CRSwNP versus CRSsNP were compared using the pooled *t*‐test; a Satterthwaite correction was applied to data where variances were unequal. Categorical data were compared using the Pearson *χ* ^2^ test. Pearson's rank correlation coefficients were calculated for pairwise data. All paired within‐subject data were analysed using the Wilcoxon‐signed rank test. Significance was accepted as *p* < 0.05. Statistical analysis was undertaken using the SAS 14.1 software programme and GraphPad Prism version 8.

## 3 RESULTS

1. The population demography is summarised in Table 1. Complete HADS data on *n* = 95 patients with CRS (*n* = 49 CRSwNP and *n* = 46 for CRSsNP) was analysed. There was no significant difference in gender, age or body mass index between the groups. Measures of CRS and asthma severity measured by the SNOT‐22, FEV_1_% predicted and ACQ‐7 did not statistically differ between the groups. AQLQ also did not differ between groups (Table 1).
2. The mean HADS scores were similar between CRSwNP and CRSsNP groups. The mean anxiety and depression scores were all at the upper limit of normal in both CRSwNP and CRSsNP (Table 2).
3. The prevalence of raised HADS anxiety and depression in CRS were 49.47 % and 38.95%, respectively. Within the CRSwNP and CRSsNP group 53.06 % and 45.66 % had raised HADS‐anxiety scores. Abnormal HADS‐depression scores were present in 40.82% and 36.95% of the CRSwNP and CRSsNP groups, respectively (Table 2). Correlations for SNOT‐22 versus HADS total was *r* = 0.59 *p* < 0.0001, HADS‐anxiety *r* = 0.56 *p* < 0.0001 and HADS‐depression *r* = 0.49 *p* < 0.0001.
4. Of the above cohort *n* = 23 patients underwent omalizumab treatment for their allergic asthma; 48% and 30.4% still had residual anxiety and depression, respectively. After 16 weeks of omalizumab treatment, HADS‐anxiety had reduced (*p* < 0.0001). A reduction in anxiety was seen both in CRSwNP and CRSsNP subtypes (*p* = 0.0042 and *p* = 0.0078, respectively). HADS‐depression scores did not improve in either group with omalizumab treatment (Table 3).
5. Both CRS and asthma severity as measured by the SNOT‐22 scores (*p* = 0.0006) and ACQ‐7 scores, respectively had improved at 16 weeks (*p* = 0.0019; Table 3).
6. The mini‐AQLQ total (*p* = 0.0003) along with individual components symptoms (*p* = 0.0007), activity (*p* = 0.0008), emotional function (*p* = 0.0009) and environmental stimuli (*p* = 0.0017) all improved at 16 weeks of treatment with omalizumab (Table 3).

**TABLE 1:** Summary of patient demography and measures of airway disease severity

Parameter	Statistic	CRSwNP	CRSsNP	*p* Value
Gender	F	28 (29.5%)	34 (35.8%)	0.0862
	M	21 (22.1%)	12 (12.6%)	
Age (years)	*N*	49	46	0.5772
	Mean (SD)	48.82 (13.12)	47.37 (12.01)	
	Minimum–maximum	20–84	21–69	
BMI (kg/m^2^)	*N*	49	46	0.1807
	Mean (SD)	27.80 (6.20)	29.59 (6.74)	
	Minimum–maximum	19–52	16–46	
SNOT‐22	*N*	40	31	0.4578
	Mean (SD)	51.63 (27.00)	46.77 (27.34)	
	Minimum–maximum	1–99	5–101	
FEV_1_%	*N*	48	45	0.9838
	Mean (SD)	78.33 (20.01)	78.42 (22.04)	
	Minimum–maximum	28–123	29–135	
ACQ‐7	*N*	42	41	
	Mean (SD)	2.92 (3.75)	2.77 (1.31)	0.8120
	Minimum–maximum	0.57–5.2	0.14–5.4	
AQLQ	*N*	45	44	0.1642
	Mean (SD)	3.94 (1.48)	3.51 (1.41)	
	Minimum–maximum	1.3–6.4	0.93–6.7	

Abbreviations: ACQ‐7, Asthma Control Questionnaire‐7; AQLQ, asthma quality of life questionnaire; BMI, body mass index; CRSsNP, chronic rhinosinusitis without nasal polyps; CRSwNP, chronic rhinosinusitis with nasal polyps; FEV_1_%, XX; SNOT‐22, sinonasal outcome test‐22.

**TABLE 2:** Summary of mental health status following appropriate medical therapy for CRS and associated asthma

Parameter	Statistic	CRSwNP	CRSsNP	*p* Value
HADS total	*N*	49	46	
	Mean (SD)	16.20 (10.09)	15.11 (10.79)	0.6102
	Minimum–maximum	0–36	0–40	
HADS anxiety	*N*	49	46	
	Mean (SD)	9.22 (5.49)	8.15 (5.83)	0.3584
	Minimum– maximum	0–20	0–20	
HADS depression	*N*	49	46	
	Mean (SD)	6.78 (5.30)	6.50 (5.20)	0.7989
	Minimum–maximum	0–18	0–20	
HADS scores ranges		0–7	8–10	11–21
CRS total	Anxiety *N* (%)	48 (50.53)	9 (9.47)	38 (40.00)
	Depression *N* (%)	58 (61.05)	14 (14.74)	23 (24.21)
CRSwNP	Anxiety *N* (%)	23 (46.94)	5 (10.20)	21 (42.86)
	Depression *N* (%)	29 (59.18)	8 (16.33)	12 (24.49)
CRSsNP	Anxiety *N* (%)	25 (54.34)	4 (8.70)	17 (36.96)
	Depression *N* (%)	29 (63.04)	6 (13.04)	11 (23.91)

Abbreviations: CRS, chronic rhinosinusitis; CRSsNP, chronic rhinosinusitis without nasal polyps; CRSwNP, chronic rhinosinusitis with nasal polyps; HADS, Hospital Anxiety and Depression Scale.

**TABLE 3:** Summary of changes in mental health status and airway disease control pre‐ and postomalizumab

CRS group	Parameter	Preomalizumab	Postomalizumab	*p* Value
CRS	*N* = 23 mean (SD)			
	Anxiety	6.78 (3.62)	3.48 (3.76)	<0.0001
	Depression	5.26 (3.37)	4.174 (3.59)	0.23
CRSwNP	*N* = 14 mean (SD)			
	Anxiety	7.21 (3.70)	3.86 (4.38)	0.0042
	Depression	6.14 (3.55)	4.786 (3.93)	0.35
CRSsNP	*N* = 9 mean (SD)			
	Anxiety	6.11 (3.59)	2.89 (2.67)	0.0078
	Depression	3.89 (2.80)	3.22 (2.95)	0.28
SNOT‐22	*N* = 23 mean (SD)	44.00 (20.73)	23.30 (19.04)	0.0006
ACQ‐7		2.10 (1.05)	1.40 (1.07)	0.0019
Mini‐AQLQ total		4.25 (1.17)	5.66 (1.36)	0.0003
Symptoms		4.200 (1.222)	5.78 (1.23)	0.0007
Activity		4.80 (1.24)	5.96 (1.37)	0.0008
Emotional function		3.99 (1.43)	5.46 (1.87)	0.0009
Environmental stimuli		4.32 (1.38)	5.52 (1.69)	0.0017

Abbreviations: ACQ‐7, Asthma Control Questionnaire‐7; AQLQ, asthma quality of life questionnaire; BMI, body mass index; CRS, chronic rhinosinusitis; CRSsNP, chronic rhinosinusitis without nasal polyps; CRSwNP, chronic rhinosinusitis with nasal polyps; SNOT‐22, sinonasal outcome test‐22.

## 4 DISCUSSION

Our findings confirm that anxiety and depression are common in patients with CRS and this is regardless of CRSwNP or CRSsNP status. We have previously shown that omalizumab treats CRS and asthma together in this group of patients.^26^ ^,^ ^27^ As far as we are aware, this is the first real‐life extension study to show that treatment with omalizumab in patients with CRS and severe asthma significantly improved anxiety but had no impact on depression. This was despite improvements in all measures of upper and lower airway disease control. The results are therefore consistent with the possibility that anxiety in CRS and asthma is closely related to current disease burden, whereas depression may be more related to factors separate to CRS and associated asthma.

Currently there is no emphasis in allergy or rhinology clinics on the recognition and treatment of anxiety and depression. ENT surgeons have little or no training in managing psychological aspects of CRS care. Our data confirm that anxiety (nearly 50% of our patients) and depression (nearly 40%) are common in patients with CRS with or without polyps. This highlights an urgent need for ENT surgeons, allergists and pulmonologists to recognise and consider direct interventions for the treatment of anxiety and particularly depression in such patients. Currently onward referral to psychiatry or treatment with anti‐depressants are almost never considered for such patients.

Despite the recognition of poor mental health status in CRS, the exact factors that predispose to anxiety and depression remain uncertain. In this real‐life study, we show that anxiety and depression, despite AMT+/‐ ESS for CRS and AMT for asthma, is still prevalent and persists to an equal degree in both CRSwNP and CRSsNP subtypes. Given this patient cohort still had high SNOT‐22 and ACQ‐7 scores, it was possible that such residual poor mental health contributed to these subjective measures of disease severity in CRS and asthma. It was also possible that the high disease scores due to the inability fully treat severe CRS and associated asthma, still maintained poor mental health status in these patients. SNOT‐22 scores strongly correlated with HADS. With further improvement in airway disease and only improvement in anxiety but not depression with omalizumab treatment, our data provides insight into how different aspects of mental health can relate to disease and others not. Systematic review of depression in CRS has failed to show any definite association with patient demography.^5^ Interestingly neither associated asthma, allergic rhinitis or other associated comorbidities such as fibromyalgia (a cause of body pain) predisposed to depression in CRS.^5^ Furthermore, previous AMT therapy or surgical intervention with functional endoscopic sinus surgery failed to improve anxiety and depression as measured with the HADS score, despite improvement in disease and disease‐specific QOL as measured by the Rhinosinusitis Disability Index.^5^

Disappointingly, the two most recent detailed phase 3 omalizumab replicate studies in CRSwNP failed to look at any changes in mental health.^28^ A previous proof of concept study of omalizumab in CRSwNP measured effect on mental health status. In *n* = 15 patients that received omalizumab, significant improvements in nasal congestion, anosmia and the AQLQ scores were seen. However, overall mental health status measured using the short form‐36 health survey and rhinosinusitis outcome measure‐31 did not improve postomalizumab.^29^ The authors did not break down the components of mental health into anxiety and depression. Our data also suggest no short‐term improvement in depression in patients with CRS and severe asthma despite omalizumab treatment. However, anxiety scores did improve in our cohort. It is possible that depression may be less amenable to change or less rapidly through airway disease treatment. Depression is often characterised with a sense of loss and thus its orientation is more towards the past, as compared to anxiety, which tends to involve a fear around events in the future. In any case, a therapeutic pathway separates to just treating airway disease that also incorporates addressing mental health is urgently needed for these patients.

The weaknesses of our study are the relatively small numbers of patients and the unblinded assessment of HADS and airway measures in the omalizumab patients. In addition, we did not consider as other possible contributing factors to depression the comorbidities commonly associated with severe asthma such as gastro‐oesophageal reflux, allergic rhinitis, associated bronchiectasis, adverse events from polypharmacy, obstructive sleep apnoea and any systemic steroid burden.

Most of our patients would have had at least four courses of high‐dose prednisolone per year for asthma and 9 of the 23 patients were on low maintenance prednisolone before and after omalizumab treatment at 16 weeks of therapy. It was only after 16 weeks of stability on omalizumab for their CRS and asthma was achieved, that reduction of prednisolone was undertaken. Clearly further detailed work is needed, but just treating mental health in CRS and asthma in a clinic setting is inadequate. Depression needs to be addressed in a more formal manner with view to more definite intervention.

## 5 CONCLUSION

The prevalence of anxiety and depression remain high in severe CRS and coassociated asthma patients despite appropriate therapy for both diseases. Further maximal treatment with omalizumab improved anxiety along with measures of upper and lower airway disease severity. Residual depression did not significantly improve and thus maybe independent of aspects of airway disease itself. Thus, a separate care pathway independent of airway disease management should be considered for depression in such patients.

## CONFLICT OF INTERESTS

Douglas S. Robinson, Harsha H. Kariyawasam have undertaken paid advisory board work for Novartis. The other authors declare no conflict of interest in relation to this manuscript. None of the authors have any financial or nonfinancial declarations in relation to the work presented in this study.

## AUTHOR CONTRIBUTIONS

All authors were involved with data set collection, interpretation and writing of the manuscript.

## References

[1] Fokkens, WJ , Lund, VJ , Hopkins, C , et al. European position paper on rhinosinusitis and nasal polyps 2020. Rhinology. 2020;58:1‐464. 10.4193/Rhin20.600 32077450

[2] Davis, GE , Yueh, B , Walker, E , et al. Psychiatric distress amplifies symptoms after surgery for chronic rhinosinusitis. Otolaryngol Head Neck Surg. 2005;132:189‐196. 15692525 10.1016/j.otohns.2004.09.135

[3] Wasan, A , Fernandez, E , Jamison, RN , Bhattacharyya, N . Association of anxiety and depression with reported disease severity in patients undergoing evaluation for chronic rhinosinusitis. Ann Otol Rhinol Laryngol. 2007;116:491‐497. 17727079 10.1177/000348940711600703

[4] Nanayakkara, JP , Igwe, C , Roberts, D , Hopkins, C . The impact of mental health on chronic rhinosinusitis symptom scores. Eur Arch Oto‐Rhino‐Laryngol. 2013;270:1361‐1364. 10.1007/s00405-012-2230-1 23095946

[5] Schlosser, RJ , Gage, SE , Kohli, P , Soler, ZM . Burden of illness: a systematic review of depression in chronic rhinosinusitis. Am J Rhinol Allergy. 2016;30:250‐256. 27456594 10.2500/ajra.2016.30.4343 PMC4953344

[6] Hsu, CL , Wang, TC , Shen, TC , et al. Risk of depression in patients with chronic rhinosinusitis: a nationwide population‐based retrospective cohort study. J Affect Disord. 2016;206:294‐299. 27643962 10.1016/j.jad.2016.09.001

[7] Caulley, L , Thavorn, K , Rudmik, L , et al. Direct costs of adult chronic rhinosinusitis by using 4 methods of estimation: results of the US Medical Expenditure Panel Survey. J Allergy Clin Immunol. 2015;136:1517‐1522. 26483176 10.1016/j.jaci.2015.08.037

[8] Kroenke, K , Spitzer, RL , Williams, JB , et al. Anxiety disorders in primary care: prevalence, impairment, comorbidity, and detection. Ann Intern Med. 2007;146:317‐325. 17339617 10.7326/0003-4819-146-5-200703060-00004

[9] Baijens, LW , Verdonschot, R , Vanbelle, S , et al. Medically unexplained otorhinolaryngological symptoms: towards integrated psychiatric care. Laryngoscope. 2015;125:1583‐1587. 25512106 10.1002/lary.25082

[10] Tomoum, MO , Klattcromwell, C , DelSignore, A , et al. Depression and anxiety in chronic rhinosinusitis. Int Forum Allergy Rhinol. 2015;5:674‐681. 25952937 10.1002/alr.21528

[11] Grenard, JL , Munjas, BA , Adams, JL , et al. Depression and medication adherence in the treatment of chronic diseases in the United States: a meta‐analysis. J Gen Intern Med. 2011;26:1175‐1182. 21533823 10.1007/s11606-011-1704-y PMC3181287

[12] Boerema, AM , Kleiboer, A , Beekman, AT , et al. Determinants of help‐seeking behavior in depression: a cross‐sectional study. BMC Psychiatr. 2016;16:78. 10.1186/s12888-016-0790-0 PMC4806501 27009062

[13] Schlosser, RJ , Hyer, JM , Smith, TL , et al. Depression‐specific outcomes after treatment of chronic rhinosinusitis. JAMA Otolaryngol Head Neck Surg. 2016;142:370‐376. 26967171 10.1001/jamaoto.2015.3810 PMC4977831

[14] Adams, KN , Schuman, TA , Ebert, CS , et al. Self‐reported anxiety and depression unchanged after endoscopic sinus surgery for chronic rhinosinusitis. Rhinology. 2018;56:234‐240. 29626844 10.4193/Rhin17.238

[15] Brandsted, R , Sindwani, R . Impact of depression on disease‐specific symptoms and quality of life in patients with chronic rhinosinusitis. Am J Rhinol. 2007;21:50‐54. 17283561 10.2500/ajr.2007.21.2987

[16] Bloechliger, M , Reinau, D , Spoendlin, J , et al. Adverse events profile of oral corticosteroids among asthma patients in the UK: cohort study with a nested case‐control analysis. Respir Res. 2018;19:75. 29699563 10.1186/s12931-018-0742-y PMC5921395

[17] Hox, V , Lourijsen, E , Jordens, A , et al. Benefits and harm of systemic steroids for short‐ and long‐term use in rhinitis and rhinosinusitis: an EAACI position paper. Clin Transl Allergy. 2020;10:1. 31908763 10.1186/s13601-019-0303-6 PMC6941282

[18] Kariyawasam, HH , Rotiroti, G . Allergic rhinitis, chronic rhinosinusitis and asthma: unravelling a complex relationship. Curr Opin Otolaryngol Head Neck Surg. 2013;21:79‐86. 23241653 10.1097/MOO.0b013e32835ac640

[19] Scott, KM , Von Korff, M , Ormel, J , et al. Mental disorders among adults with asthma: results from the World Mental Health Survey. Gen Hosp Psychiatr. 2007;29:123‐133. 10.1016/j.genhosppsych.2006.12.006 PMC1913936 17336661

[20] Schlosser, RJ , Storck, K , Cortese, BM , et al. Depression in chronic rhinosinusitis: a controlled cohort study. Am J Rhinol Allergy. 2016;30:128‐133. 10.2500/ajra.2016.30.4290 PMC5554332 26980393

[21] Zigmond, AS , Snaith, RP . The Hospital Anxiety and Depression Scale. Acta Psychiatr Scand. 1983;67:361‐370. 6880820 10.1111/j.1600-0447.1983.tb09716.x

[22] Toma, S , Hopkins, C . Stratification of SNOT‐22 scores into mild, moderate or severe and relationship with other subjective instruments. Rhinology. 2016;54:129‐133. 27017484 10.4193/Rhino15.072

[23] Juniper, EF , O'Byrne, PM , Guyatt, GH , et al. Development and validation of a questionnaire to measure asthma control. Eur Respir J. 1999;14:902‐907. 10573240 10.1034/j.1399-3003.1999.14d29.x

[24] Juniper, EF , Guyatt, GH , Epstein, RS , et al. Evaluation of impairment of health related quality of life in asthma: development of a questionnaire for use in clinical trials. Thorax. 1992;47:76‐83. 1549827 10.1136/thx.47.2.76 PMC463574

[25] Juniper, EF , Guyatt, GH , Cox, FM , et al. Development and validation of the mini asthma quality of life questionnaire. Eur Respir J. 1999;14:32‐38. 10489826 10.1034/j.1399-3003.1999.14a08.x

[26] Bidder, T , Sahota, J , Rennie, C , et al. Omalizumab treats chronic rhinosinusitis with nasal polyps and asthma together—a real life study. Rhinology. 2018;56:42‐45. 29288573 10.4193/Rhino17.139

[27] Sahota, J , Bidder, T , Livingston, R , et al. Chronic rhinosinusitis and omalizumab: eosinophils not IgE predict treatment response in real‐life (2018).

[28] Gevaert, P , Omachi, TA , Corren, J , et al. Efficacy and safety of omalizumab in nasal polyposis: two randomized phase III trials. J Allergy Clin Immunol. 2020;146:595–605. 32524991 10.1016/j.jaci.2020.05.032

[29] Gevaert, P , Calus, L , Van, ZT , et al. Omalizumab is effective in allergic and nonallergic patients with nasal polyps and asthma. J Allergy Clin Immunol. 2013;131:110‐116. 23021878 10.1016/j.jaci.2012.07.047

